# Preoperative differentiation of spinal tuberculosis, pyogenic, and brucellar spondylitis: a multimodal machine learning study across five centers

**DOI:** 10.3389/fcimb.2026.1925786

**Published:** 2026-09-16

**Authors:** Xingyu Duan, Linan Wang, Yichao Fan, Jiaxing Wang, Qinghong Huang, Hekun Liu, Lili Deng, Wensheng Liao, Ningkui Niu

**Affiliations:** 1 Department of Orthopedics, General Hospital of Ningxia Medical University, Yinchuan, China; 2 First Clinical Medical College, Ningxia Medical University, Yinchuan, China; 3 Department of Orthopedics, Zhoukou Central Hospital, Zhoukou, China; 4 Department of Tuberculosis, Henan Infectious Disease Hospital (The Sixth People’s Hospital of Zhengzhou), Zhengzhou, China; 5 Department of General Practice, The First People’s Hospital of Zhengzhou, Zhengzhou, China; 6 Department of Spinal and Spinal Cord Surgery, Henan Provincial People’s Hospital, Zhengzhou, China; 7 Ningxia Key Laboratory of Clinical Pathogenic Microorganisms, General Hospital of Ningxia Medical University, Yinchuan, China

**Keywords:** explainable artificial intelligence, external validation, infectious spondylitis, machine learning, multimodal fusion

## Abstract

**Background:**

Infectious spondylitis, encompassing spinal tuberculosis (STB), pyogenic spondylitis (PS), and brucellar spondylitis (BS), is difficult to diagnose preoperatively because the three entities present with overlapping clinical features. Differentiation is often delayed by prolonged culture turnaround times and the unavailability of serological testing.

**Methods:**

In this multicenter retrospective diagnostic study at five tertiary hospitals in China (2015–2023), patients with STB (n = 1,000), PS (n = 579), and BS (n = 255) were enrolled and assigned to derivation (n = 1,439) and external validation (n = 395) cohorts. We developed and validated a multimodal machine learning model that integrates routine preoperative clinical and laboratory variables with radiomic features extracted from non-contrast MRI. Unimodal, early fusion, and late fusion architectures were compared. Pathogen-specific serological tests were excluded from the primary model to avoid data leakage. We evaluated discrimination, calibration, clinical utility, and incremental value; SHAP analysis provided case-level interpretability.

**Results:**

The late fusion LightGBM model achieved the highest external validation performance (macro-averaged AUC 0.852, 95% CI 0.822–0.882). Radiomic integration provided significant incremental value over the clinical-only model (ΔAUC 0.080; NRI 0.398; IDI 0.072). Calibration was good (Brier score 0.158), and decision curve analysis demonstrated positive net benefit across clinically relevant thresholds. SHAP analysis revealed class-specific, biologically plausible predictors: low serum albumin and elevated T1W GLCM contrast for STB; high CRP and NLR for PS; and residence in an endemic region for BS. Secondary analysis showed that adding serological tests yielded only modest incremental improvement (ΔAUC 0.042).

**Conclusions:**

This preoperative multimodal explainable AI model achieves accurate and interpretable differentiation of STB, PS, and BS using only routine clinical data and non-contrast MRI, although its sensitivity for BS remains limited and prospective validation is warranted. It may serve as a clinically actionable decision-support tool for preoperative triage, particularly when serological confirmation is pending or unavailable.

## Graphical Abstract

**Figure:**
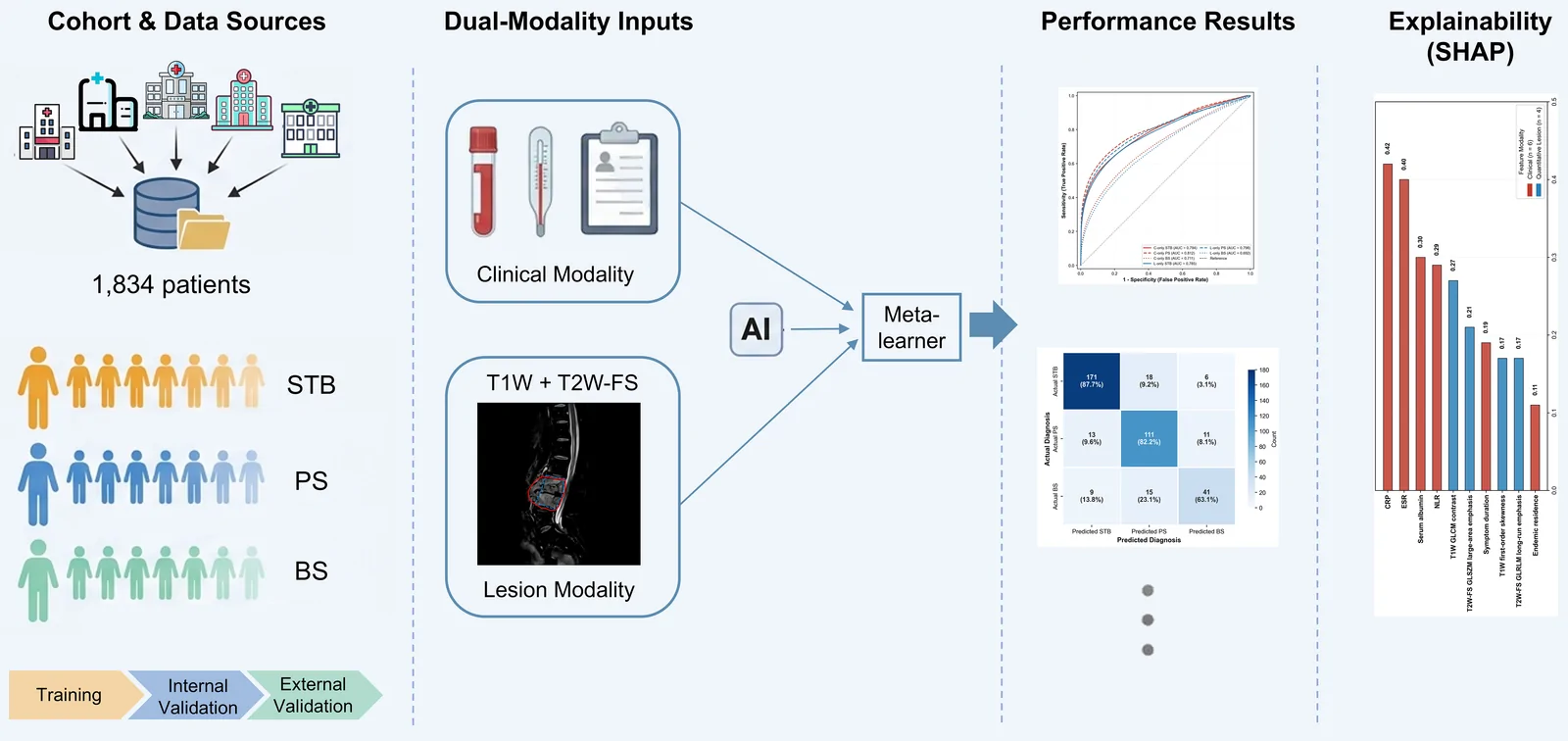

## Introduction

Infectious spondylitis, encompassing spinal tuberculosis (STB), pyogenic spondylitis (PS), and brucellar spondylitis (BS), is difficult to diagnose (Tanishima et al., 2024; Khanna and Sabharwal, 2019; Jung et al., 2004). STB accounts for roughly half of all skeletal tuberculosis worldwide (Thammaroj et al., 2014); PS progresses rapidly and may cause sepsis and irreversible deformity (Kramer et al., 2025); and BS remains endemic in agricultural regions where unpasteurized dairy is consumed (Wang K. et al., 2025). Although the three diseases differ pathophysiologically, with chronic granulomatous inflammation in STB, acute neutrophilic suppuration in PS, and subacute inflammation in BS, they present with overlapping clinical features, which often leads to delayed diagnosis and inappropriate empiric therapy (Ren et al., 2024; Ma et al., 2025; Hu et al., 2022).

Current diagnosis depends on clinical evaluation, laboratory tests, culture, and molecular assays, yet each approach has important limitations. Intraoperative tissue culture remains the gold standard but is time-consuming and has limited sensitivity for Mycobacterium tuberculosis (Jin et al., 2023; Zhang et al., 2025). Molecular methods are expensive and require specialized infrastructure (Ma et al., 2024; Jain et al., 2023). Pathogen-specific serological tests, including interferon-gamma release assays such as T-SPOT.TB, are useful confirmatory tools but are often unavailable when surgical decisions must be made.

Machine learning offers a promising way to augment clinical decision-making. Existing spondylitis models, however, have been predominantly unimodal, relying on either clinical variables or radiological features alone, and have been limited by single-center designs and lack of external validation (Yao et al., 2023; Mo et al., 2026; Hu et al., 2025). Few studies have addressed the three-class differential diagnosis of STB, PS, and BS (Gao et al., 2025). In addition, the black-box nature of many models has hindered clinical adoption because clinicians need transparent, interpretable predictions to justify treatment decisions.

Radiomics extracts high-dimensional quantitative features from medical images, providing complementary information that captures tissue-level pathophysiological heterogeneity (Qin et al., 2025; Yimit et al., 2025). When combined with clinical data, radiomic signatures may reflect distinct inflammatory patterns—such as caseous necrosis in STB and abscess formation in PS—that are not discernible to the naked eye. The optimal strategy for fusing these modalities and the biological interpretability of the resulting biomarkers remain to be determined.

To address these gaps, we conducted a multicenter retrospective study to develop and externally validate a preoperative multimodal machine learning model for the differential diagnosis of STB, PS, and BS. The model integrates routine preoperative clinical and laboratory variables with radiomic features derived from non-contrast MRI. Pathogen-specific serological tests were deliberately excluded from the primary model. We compared unimodal, early fusion, and late fusion architectures and used SHAP to provide clinically actionable, case-level interpretability.

## Methods

### Study design and cohort

This multicenter retrospective diagnostic study enrolled patients who underwent surgery for suspected infectious spondylitis between January 2015 and December 2023 at five tertiary hospitals in two provinces in northwestern and central China. The participating centers were: (i) the General Hospital of Ningxia Medical University (Yinchuan, Ningxia), (ii) Henan Provincial People’s Hospital (Zhengzhou, Henan), (iii) Henan Infectious Disease Hospital (Zhengzhou, Henan), (iv) The First People’s Hospital of Zhengzhou (Zhengzhou, Henan), and (v) Zhoukou Central Hospital (Zhoukou, Henan). The study protocol was approved by the Institutional Ethics Committee at each center, and informed consent was waived because of the retrospective design. The study was conducted in accordance with the Declaration of Helsinki and reported according to the TRIPOD+AI (2024) guidelines (Collins et al., 2024). The patient flowchart is presented in **Figure 1**.

**Figure 1:**
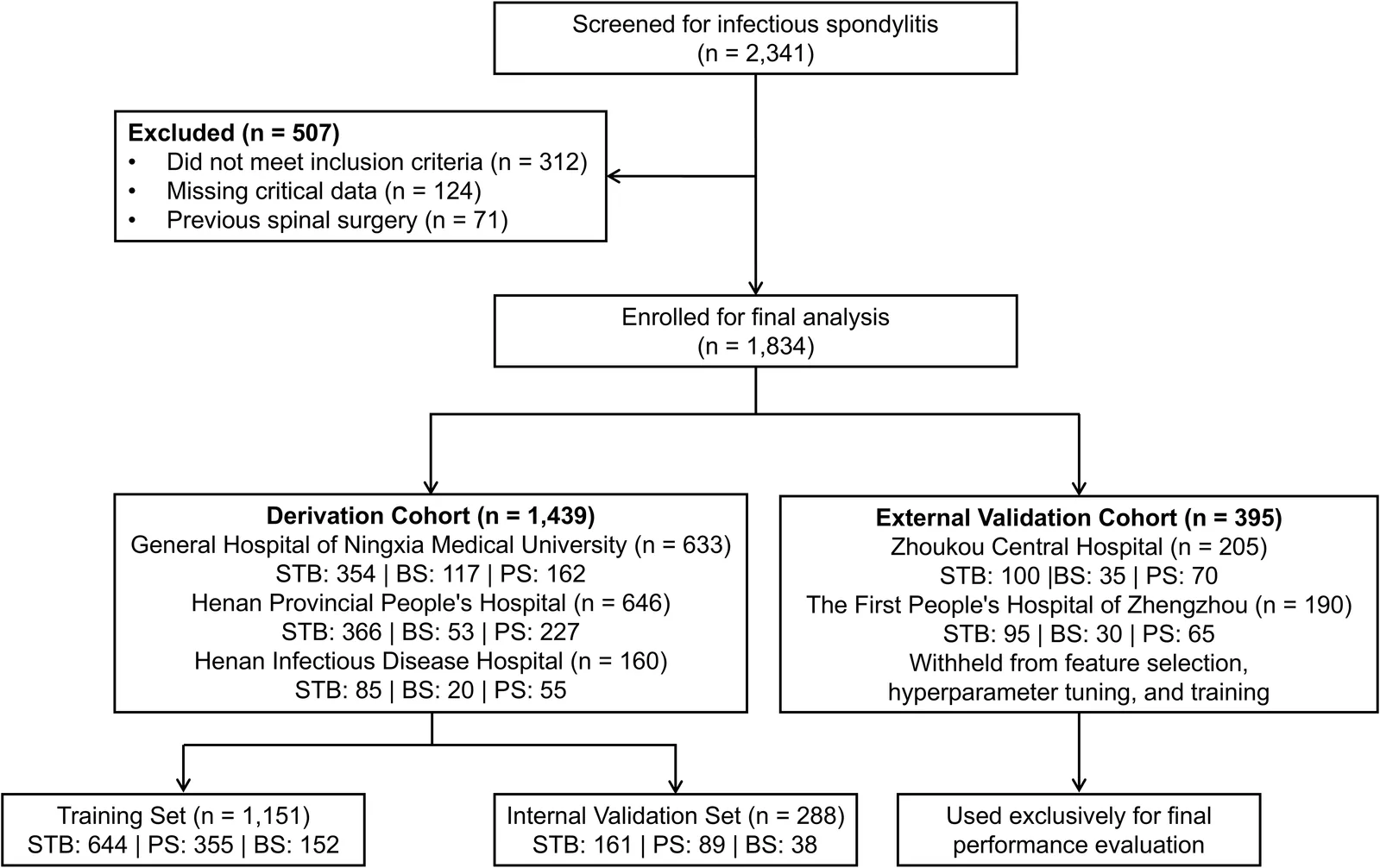
Patient flowchart for the multicenter development and external validation of the multimodal machine learning model.

Patients were classified retrospectively into three mutually exclusive diagnostic groups using a predefined, rigorously applied reference standard. The five-center dataset was split by institution into a derivation cohort (three centers, n = 1,439) and an external validation cohort (two centers, n = 395). Within the derivation cohort, a stratified 8:2 random split generated a training set (n = 1,151) and an internal validation set (n = 288), preserving class proportions. The external validation cohort was not used for feature selection, hyperparameter tuning, model training, or threshold determination. The derivation centers collected data from 2015 to 2023, whereas the external validation centers collected data from 2019 to 2023; no patient overlap existed between cohorts. Data collection and reference-standard adjudication in the external validation cohort were completed before the model development pipeline, which included feature selection, algorithm choice, and hyperparameter tuning, was finalized in the derivation cohort, ensuring that the external validation constituted a fully independent evaluation.

The center-level distribution of the final enrolled cohort is detailed in **Table 1**. In total, 1,834 patients were enrolled after applying inclusion and exclusion criteria. The derivation cohort comprised 633 patients from the General Hospital of Ningxia Medical University (354 STB, 117 BS, 162 PS), 646 patients from Henan Provincial People’s Hospital (366 STB, 53 BS, 227 PS), and 160 patients from Henan Infectious Disease Hospital (85 STB, 20 BS, 55 PS). The external validation cohort comprised 205 patients from Zhoukou Central Hospital (100 STB, 35 BS, 70 PS) and 190 patients from The First People’s Hospital of Zhengzhou (95 STB, 30 BS, 65 PS).

**Table 1:** Baseline characteristics of the study cohort.

Characteristic	Training set (n = 1,151)	Internal validation (n = 288)	External validation (n = 395)	P-value^a^	STB (n = 1,000)	PS (n = 579)	BS (n = 255)	P-value^b^
Demographics								
Age, years, mean ± SD	51.8 ± 13.0	51.0 ± 12.8	51.7 ± 13.1	0.68	51.1 ± 12.4	55.9 ± 13.1	44.3 ± 11.2	<0.001
Male, n (%)	693 (60.2)	177 (61.5)	260 (65.8)	0.14	587 (58.7)	354 (61.1)	189 (74.1)	<0.001
BMI, kg/m², mean ± SD	22.7 ± 3.4	22.5 ± 3.4	22.5 ± 3.4	0.61	21.8 ± 3.1	24.1 ± 3.4	22.5 ± 3.1	<0.001
Endemic region, n (%)	419 (36.4)	111 (38.5)	152 (38.5)	0.67	276 (27.6)	197 (34.0)	209 (82.0)	<0.001
Symptoms and comorbidities								
Symptom duration, days, median (IQR)	47 (23–92)	50 (21–96)	43 (22–87)	0.4	75 (46–125)	18 (11–32)	41 (24–71)	<0.001
Fever, n (%)	675 (58.6)	167 (58.0)	230 (58.2)	0.97	473 (47.3)	423 (73.1)	176 (69.0)	<0.001
Night sweats, n (%)	466 (40.5)	116 (40.3)	158 (40.0)	0.99	609 (60.9)	43 (7.4)	88 (34.5)	<0.001
Weight loss, n (%)	447 (38.8)	122 (42.4)	159 (40.3)	0.53	529 (52.9)	108 (18.7)	91 (35.7)	<0.001
Neurological deficit, n (%)	169 (14.7)	37 (12.8)	53 (13.4)	0.66	129 (12.9)	84 (14.5)	46 (18.0)	0.1
Diabetes mellitus, n (%)	135 (11.7)	41 (14.2)	38 (9.6)	0.18	110 (11.0)	71 (12.3)	33 (12.9)	0.6
Laboratory indicators								
WBC, ×10^9^/L, median (IQR)	8.3 (6.7–10.9)	8.2 (6.8–10.9)	8.5 (6.7–11.2)	0.67	7.4 (6.2–8.7)	12.1 (9.6–15.5)	7.8 (6.3–9.5)	<0.001
Neutrophils, ×10^9^/L, median (IQR)	5.9 (4.4–8.9)	6.0 (4.3–8.8)	6.1 (4.4–8.9)	0.5	4.9 (3.8–6.1)	10.4 (7.9–13.6)	5.6 (4.1–7.3)	<0.001
Lymphocytes, ×10^9^/L, median (IQR)	1.2 (0.9–1.7)	1.3 (0.9–1.8)	1.2 (0.9–1.7)	0.41	1.5 (1.1–2.0)	0.8 (0.6–1.1)	1.2 (0.9–1.6)	<0.001
Hemoglobin, g/L, mean ± SD	118.6 ± 19.9	118.2 ± 19.5	118.9 ± 20.1	0.93	112.1 ± 17.0	128.9 ± 20.7	120.5 ± 18.4	<0.001
Platelets, ×10^9^/L, median (IQR)	273 (213–345)	273 (206–337)	276 (214–350)	0.47	243 (193–308)	325 (261–403)	288 (222–351)	<0.001
CRP, mg/L, median (IQR)	56.8 (34.2–91.5)	54.6 (34.8–90.4)	56.1 (30.9–90.6)	0.66	41.8 (28.1–62.0)	105.6 (75.9–149.9)	43.2 (30.3–65.4)	<0.001
ESR, mm/h, median (IQR)	66.1 (44.7–98.7)	67.0 (46.7–97.4)	69.0 (47.2–100.9)	0.51	57.6 (40.1–83.0)	92.2 (66.5–121.3)	54.4 (37.2–81.6)	<0.001
Albumin, g/L, mean ± SD	37.2 ± 5.5	37.7 ± 5.2	37.7 ± 5.3	0.24	35.3 ± 4.8	40.4 ± 5.2	38.7 ± 4.4	<0.001
Prealbumin, mg/L, mean ± SD	168.5 ± 44.3	166.4 ± 43.1	170.2 ± 45.3	0.55	153.1 ± 37.8	189.6 ± 45.9	181.1 ± 40.8	<0.001
ALT, U/L, median (IQR)	30.2 (19.7–45.8)	31.1 (21.8–47.6)	28.9 (18.4–43.8)	0.18	33.9 (22.0–50.0)	25.7 (16.6–38.1)	27.9 (19.5–40.0)	<0.001
AST, U/L, median (IQR)	33.2 (21.5–48.7)	31.9 (22.6–49.7)	32.6 (22.7–46.1)	0.89	35.6 (24.8–53.6)	29.3 (19.7–43.3)	28.9 (19.3–43.6)	<0.001
Creatinine, μmol/L, median (IQR)	73.0 (59.4–90.1)	72.4 (56.3–90.8)	71.6 (59.0–89.1)	0.85	69.3 (56.0–86.1)	80.3 (63.8–97.9)	71.5 (55.9–86.8)	<0.001
Inflammatory-nutritional indices								
NLR, median (IQR)	4.6 (2.8–9.4)	4.6 (2.7–8.9)	5.0 (3.1–8.9)	0.34	3.2 (2.3–4.6)	13.3 (8.8–18.5)	4.6 (3.0–6.8)	<0.001
PLR, median (IQR)	221.7 (141.7–362.0)	218.0 (127.3–343.0)	225.4 (145.4–362.7)	0.27	162.0 (110.6–233.1)	410.5 (275.5–580.1)	228.4 (160.8–325.3)	<0.001
LMR, median (IQR)	2.8 (1.8–4.4)	2.9 (1.8–4.8)	2.7 (1.8–4.4)	0.76	3.5 (2.3–5.4)	1.9 (1.2–2.8)	2.8 (1.7–4.1)	<0.001
SII, median (IQR)	1,271 (702–2,922)	1,181 (597–2,800)	1,415 (705–2,778)	0.25	782 (512–1,227)	4,390 (2,650–6,590)	1,321 (811–1,950)	<0.001
PNI, mean ± SD	44.0 ± 5.8	44.7 ± 5.5	44.4 ± 5.7	0.14	43.5 ± 5.9	44.9 ± 5.6	45.3 ± 5.2	<0.001
CAR, median (IQR)	1.53 (0.97–2.48)	1.52 (0.91–2.37)	1.46 (0.92–2.45)	0.5	1.18 (0.80–1.80)	2.59 (1.85–3.73)	1.10 (0.76–1.78)	<0.001
Quantitative lesion characteristics								
Lesion volume, cm³, median (IQR)	14.2 (5.9–35.7)	13.9 (5.4–32.4)	13.0 (5.0–36.6)	0.68	17.9 (7.7–44.0)	12.6 (5.0–28.8)	6.0 (2.5–16.3)	<0.001
Involved segments, median (IQR)	2.0 (1.0–2.0)	2.0 (1.0–2.0)	2.0 (1.0–2.0)	0.92	2.0 (1.0–3.0)	2.0 (1.0–2.0)	1.0 (1.0–2.0)	<0.001
T1W GLCM contrast, mean ± SD	0.037 ± 0.011	0.037 ± 0.011	0.037 ± 0.012	0.76	0.041 ± 0.011	0.032 ± 0.009	0.029 ± 0.008	<0.001
T2W-FS entropy, mean ± SD	5.54 ± 0.65	5.59 ± 0.65	5.56 ± 0.65	0.51	5.43 ± 0.62	5.78 ± 0.68	5.53 ± 0.57	<0.001
T2W-FS GLSZM large-area emphasis, ×10^-3^	0.089 (0.045–0.181)	0.087 (0.042–0.154)	0.082 (0.045–0.167)	0.44	0.115 (0.059–0.214)	0.067 (0.034–0.134)	0.051 (0.028–0.106)	<0.001
T1W original shape Sphericity, mean ± SD	0.62 ± 0.14	0.63 ± 0.14	0.63 ± 0.14	0.53	0.58 ± 0.12	0.67 ± 0.14	0.72 ± 0.13	<0.001

^a^ P-value across dataset partitions;

^b^ P-value across diagnostic groups (omnibus one-way ANOVA, Kruskal–Wallis, or χ² test, as appropriate). GLSZM large-area emphasis values are presented in units of 10⁻³ for readability. IQR, interquartile range; NLR, neutrophil-to-lymphocyte ratio; PLR, platelet-to-lymphocyte ratio; LMR, lymphocyte-to-monocyte ratio; SII, systemic immune-inflammation index; PNI, prognostic nutritional index; CAR, CRP-to-albumin ratio; GLCM, gray-level co-occurrence matrix; GLSZM, gray-level size zone matrix; T1W, T1-weighted; T2W-FS, T2-weighted fat-saturation.

### Reference standard and diagnostic groups

A panel of three senior orthopedic surgeons, two infectious disease specialists, and one musculoskeletal radiologist, all blinded to the machine learning predictions, retrospectively adjudicated the final diagnosis for each patient using predefined, hierarchically ranked criteria.

Spinal Tuberculosis (STB): Definite STB required positive Mycobacterium tuberculosis culture or Xpert MTB/RIF assay from lesion tissue, pus, or intraoperative biopsy. Probable STB was defined as a compatible clinical-radiological syndrome with positive interferon-gamma release assay (IGRA) or T-SPOT.TB, exclusion of other pathogens, and favorable response to standard anti-tuberculosis therapy for at least 6 months. Histopathological demonstration of caseating granulomas with Langhans giant cells was considered supportive but not sufficient alone for definite diagnosis, given the known overlap with other granulomatous conditions and sampling variability in spinal lesions.

Pyogenic Spondylitis (PS): Definite PS required positive bacterial culture from blood or intraoperative tissue or biopsy, or positive next-generation sequencing (NGS) identifying a pathogenic bacterium. Histopathological evidence of acute suppurative inflammation with neutrophilic infiltration and abscess formation was also accepted. Probable PS required a compatible clinical-radiological syndrome with negative or inconclusive microbiological results, documented favorable response to targeted antibiotic therapy, and exclusion of tuberculosis and brucellar infection.

Brucellar Spondylitis (BS): Definite BS required a positive standard agglutination test (SAT) titer ≥1:160 or positive Rose Bengal test with compatible clinical-radiological findings, or positive culture or PCR from tissue or blood. Probable BS was defined as seropositivity with a compatible MRI pattern and exclusion of other etiologies in an endemic region.

Exclusion criteria included: age <18 years; less than 12 months of follow-up; concurrent HIV infection or other immunodeficiency states; co-infection with two or more pathogens; previous spinal surgery at the same level; incomplete preoperative MRI of insufficient quality for feature extraction; missing any critical preoperative clinical or laboratory data; and inability to establish a definite or probable diagnosis per the above criteria. Patients with non-infectious Modic changes or degenerative endplate abnormalities were excluded if they did not meet the infectious diagnostic criteria. After these exclusions, all remaining patients had complete data; no imputation was required.

### Feature extraction

Lesion Characterization Features. PyRadiomics v3.0.1 was used to extract 2,632 quantitative features from each segmented ROI across T1W and T2W-FS sequences (1,316 per sequence). The feature set included: (i) first-order statistics (n = 504; 18 per image); (ii) shape-based features (n = 28; 14 per sequence); and (iii) texture features (n = 2,100; 75 per image): GLCM (24 features, computed from three-dimensional co-occurrence matrices aggregated over 13 directions), GLRLM (16), GLSZM (16), NGTDM (5), and GLDM (14). All texture features were computed with a fixed bin width of 25 and a Chebyshev distance of 1. Additional filters were applied: wavelet decomposition (8 sub-bands), Laplacian of Gaussian (LoG, sigma = 1.0, 2.0, 3.0 mm), and square and square-root transformations.

Clinical and Laboratory Predictors. Clinical data were extracted from the 72 hours before surgery and included demographics [age, sex, body mass index (BMI)*, residence in a brucellosis-endemic vs. non-endemic region]; symptoms and comorbidities [duration of symptoms (days), fever, night sweats, weight loss, neurological deficit (ASIA grade), diabetes mellitus]; laboratory indicators [complete blood count (white blood cells, neutrophils, lymphocytes, monocytes, hemoglobin, platelets), erythrocyte sedimentation rate (ESR), C-reactive protein (CRP), albumin, prealbumin, total protein, alanine aminotransferase (ALT), aspartate aminotransferase (AST), creatinine, prothrombin time, and D-dimer]; and inflammatory-nutritional composite indices [neutrophil-to-lymphocyte ratio (NLR), platelet-to-lymphocyte ratio (PLR), lymphocyte-to-monocyte ratio (LMR), systemic immune-inflammation index (SII), prognostic nutritional index (PNI), and CRP-to-albumin ratio (CAR)].

Pathogen-specific tests—including the serological assays T-SPOT.TB, IGRA (QuantiFERON-TB Gold), and Brucella standard agglutination test (SAT), as well as microbiological acid-fast bacilli smear—were deliberately excluded from the primary model because they are components of the reference standard for STB and BS diagnosis. Including them as predictors would introduce data leakage and inflate model performance artificially. Their incremental diagnostic value was evaluated separately in a secondary sensitivity analysis.

### MRI acquisition and lesion segmentation

All patients underwent preoperative spinal MRI on 1.5T or 3.0T scanners (Siemens Magnetom, GE Discovery, or Philips Ingenia). MRI protocols were standardized across institutions: T1-weighted (T1W) spin-echo (TR 400–600 ms, TE 10–15 ms) and T2-weighted fat-saturation (T2W-FS) or short-tau inversion recovery (STIR) sequences (TR 3000–5000 ms, TE 60–120 ms, plus TI 150–180 ms for STIR) in the sagittal plane. Slice thickness was ≤4 mm with no interslice gap. Only sagittal T1W and T2W-FS/STIR sequences were used for analysis, as these are universally available and provide the highest tissue contrast for spondylitis lesion characterization.

Contrast-enhanced T1-weighted sequences were not included. Because this was a retrospective multicenter study spanning 2015–2023, contrast-enhanced MRI was not performed routinely at all centers, particularly during the earlier years or at resource-limited sites. Gadolinium administration was at the discretion of the treating physician, resulting in substantial heterogeneity and missing data for contrast-enhanced sequences. To ensure methodological consistency and avoid selection bias, we restricted radiomic feature extraction to non-contrast T1W and T2W-FS/STIR sequences, which were available for all patients.

Images were preprocessed using Python 3.10: (1) resampling to an isotropic voxel spacing of 1.0 × 1.0 × 1.0 mm³ using B-spline interpolation (SimpleITK v2.3.0) to harmonize spatial resolution across scanners; (2) intensity normalization using Z-score standardization within the region of interest to reduce inter-scanner drift.

Lesion regions of interest (ROIs) were manually delineated on sagittal T1W and T2W-FS images by two radiologists (5 and 10 years of experience in musculoskeletal MRI) using 3D Slicer v5.2.2. Segmentation was performed as a three-dimensional volumetric delineation on a slice-by-slice basis in the sagittal plane, rather than on a single representative slice, to ensure accurate extraction of shape-based features such as volume, surface area, and sphericity. The ROI encompassed the affected vertebral bodies, intervertebral discs, and associated paravertebral or epidural abscesses if present. Inter-observer segmentation agreement was assessed in 50 randomly selected cases from the derivation cohort (approximately 3.5%), a sample size consistent with standard radiomics reproducibility studies. The mean Dice similarity coefficient (DSC) for vertebral body and disc segmentation was >0.90, and for abscess components was >0.85. Features were independently extracted from both sets of segmentations; quantitative features with an intraclass correlation coefficient (ICC) < 0.80 were deemed unstable and excluded from downstream analysis, thereby minimizing the impact of segmentation variability on model performance.

### Feature selection and dimensionality reduction

Feature selection followed a three-stage pipeline applied strictly within the training set.

Stage I: Stability and Redundancy Filtering. Quantitative lesion features with intraclass correlation coefficient (ICC) < 0.80 were excluded. Highly correlated features (Pearson |r| > 0.90 or Spearman |ρ| > 0.90) were identified, and the feature with lower mutual information with the outcome was removed from each pair.

Stage II: Univariate Screening. Clinical variables were compared across the three diagnostic groups using one-way ANOVA for normally distributed continuous variables, the Kruskal–Wallis test for non-normally distributed continuous variables, and the χ² test or Fisher’s exact test for categorical variables. Variables with P < 0.05 were retained. Quantitative lesion features were screened using ANOVA with false discovery rate (FDR) correction (Benjamini-Hochberg, q < 0.05).

Stage III: Modality-Specific Multivariable Selection. Clinical features were selected using least absolute shrinkage and selection operator (LASSO) regression with multinomial logistic loss and 10-fold cross-validation; the optimal regularization parameter (λ) was determined by the 1-standard-error (1-SE) rule. Quantitative lesion features were ranked using the minimum redundancy maximum relevance (mRMR) algorithm, and the top 20 features were retained. This produced two modality-specific feature subsets: Clinical (C) and Quantitative Lesion (L).

### Model development and fusion architecture

We evaluated four supervised machine learning algorithms for multiclass discrimination: LightGBM (Ke et al., 2017), XGBoost (Chen and Guestrin, 2016), Random Forest, and Multinomial Logistic Regression (LR). We configured all models for three-class classification (STB vs. PS vs. BS). Because the cohort was imbalanced (BS accounted for 13.9%), we compared two strategies:

1. Class-weighted loss (primary analysis):
(1) LightGBM: *class_weight = 'balanced'*; XGBoost: per-sample weights via sklearn's *compute_sample_weight('balanced', y_train)*.
(2) RF and LR: *class_weight = 'balanced'*
2. SMOTE (sensitivity analysis): We synthetically oversampled the minority class (BS) in the training set to match the majority class.

We reported class-specific performance metrics for BS (sensitivity, PPV, F1-score) separately so that poor minority-class performance would not be hidden behind macro-averages. We optimized hyperparameters using stratified 5-fold cross-validation within the training set and Bayesian optimization (Optuna v3.4.0, 200 trials), with macro-averaged AUC as the objective function.

We compared three fusion architectures against unimodal baselines:

1. Unimodal Baselines (C-only, L-only): Models trained exclusively on clinical or quantitative lesion features.
2. Early Fusion (EF-C+L): After modality-specific selection (Stage III), we concatenated features from the Clinical (C) and Quantitative Lesion (L) subsets into a single vector. We applied no further dimensionality reduction, so both modalities retained full representation.
3. Late Fusion (LF-C+L): We independently trained unimodal models for each modality, concatenated their predicted class probabilities (softmax outputs), and fed these into a meta-learner (multinomial logistic regression with L2 regularization) to produce the final diagnostic probability. We trained the meta-learner on out-of-fold predictions from 5-fold cross-validation to avoid information leakage.

### Model evaluation and validation strategy

Model performance was evaluated in the internal validation set (for model selection) and the external validation set (for final, unbiased performance reporting). The following metrics were computed for each class and macro-averaged across classes.

Discrimination. The primary metric was the Hand and Till M-statistic (Hand and Till, 2001) (macro-averaged one-vs-one multiclass AUC-ROC), with 95% confidence intervals from 1,000 bootstrap resamplings. One-vs-rest (OvR) AUCs were also computed for each class (STB vs. PS+BS; PS vs. STB+BS; BS vs. STB+PS) to support binary clinical decision rules. Pairwise AUC comparisons were performed using 1,000-repetition bootstrap resampling to estimate confidence intervals and P values, given the limitations of the DeLong test in multiclass settings.

Calibration. Calibration curves, intercept, slope, and multiclass Brier score.

Classification Metrics. Accuracy, macro-averaged sensitivity, specificity, positive predictive value (PPV), negative predictive value (NPV), and F1-score. Class-specific confusion matrices were generated.

Clinical Utility. Decision curve analysis (DCA) was performed to quantify net benefit across clinically plausible threshold probabilities (0–100%), compared with treat-all and treat-none strategies.

Incremental Value of Fusion. The incremental value of adding quantitative lesion features to clinical data was quantified by comparing the best multimodal model with the best unimodal clinical model: ΔAUC with bootstrap significance; category-free net reclassification improvement (NRI) adapted to the multiclass setting per Li et al. (2013) and Pencina et al. (2011); integrated discrimination improvement (IDI) per Pencina et al. (2008); and relative utility (RU) at clinically relevant thresholds. Bootstrap resampling (1,000 repetitions) was used to estimate standard errors and 95% confidence intervals.

Secondary Sensitivity Analysis: Incremental Value of Serological Tests. In a secondary analysis, pathogen-specific serological tests (T-SPOT.TB/IGRA for STB and Brucella SAT for BS) were added to the primary model. The incremental discriminative value was quantified using ΔAUC, NRI, and IDI.

### Model interpretability

SHAP Analysis. SHapley Additive exPlanations (SHAP) values were computed for the best-performing tree-based model in the external validation set to ensure unbiased estimation of feature contributions. SHAP summary plots, four-panel dependence plots, and two-panel waterfall decomposition plots were generated to visualize global feature importance (mean absolute SHAP value), directional effects of individual features on class probabilities, and case-level attribution for representative patients.

Lesion Feature Interpretation. The top 10 quantitative lesion features by SHAP importance were correlated with their anatomical and pathological meanings to connect quantitative features with pathophysiological processes.

### Statistical analysis and scanner heterogeneity control

Descriptive statistics are presented as mean ± standard deviation (SD) for normally distributed continuous variables, median (interquartile range, IQR) for skewed continuous variables, and frequencies (percentages) for categorical variables. Normality was assessed using the Shapiro-Wilk test. Between-group comparisons were performed using one-way ANOVA (or Kruskal-Wallis test with Dunn’s *post-hoc* test and Bonferroni correction) for continuous variables and χ² or Fisher’s exact test for categorical variables. All feature selection and model training were confined to the training set. Statistical significance was set at two-sided P < 0.05.

*Post-hoc* power analysis for the BS subgroup was performed using the Hanley–McNeil method (Hanley and McNeil, 1982) to estimate the standard error (SE) of the one-vs-rest AUC in the external validation set; statistical power was calculated for detecting AUC values exceeding prespecified clinical thresholds (0.70 and 0.75) at a one-sided α of 0.05. Events per variable (EPV) for the BS class in the training set was calculated as a heuristic measure of sample-size adequacy.

Scanner Heterogeneity Control. To mitigate potential confounding from multi-scanner acquisition (1.5T vs. 3.0T; Siemens, GE, Philips), scanner manufacturer and field strength were recorded for each patient. Multivariable linear regression was used to evaluate whether these technical factors were associated with the top-ranked quantitative lesion features; no significant associations were observed after FDR correction. The external validation centers used different scanner models than the derivation centers, ensuring that generalizability was assessed across both patient populations and imaging platforms. All analyses were performed using Python 3.10 (scikit-learn, LightGBM, XGBoost, PyRadiomics, SHAP, Optuna) and R 4.3.0 (pROC, rms, rmda, CalibrationCurves).

## Results

### Patient enrollment, dataset partitioning, and baseline characteristics

A total of 2,341 patients who underwent surgery for suspected infectious spondylitis were screened across five participating centers between January 2015 and December 2023. After applying inclusion and exclusion criteria, 1,834 patients were enrolled and allocated to a derivation cohort (n = 1,439) and an independent external validation cohort (n = 395). Within the derivation cohort, a stratified 8:2 random split generated a training set (n = 1,151) and an internal validation set (n = 288). The three diagnostic classes were distributed as STB (n = 1,000, 54.5%), PS (n = 579, 31.6%), and BS (n = 255, 13.9%). Stratified splitting preserved class proportions between the training and internal validation sets, whereas the external validation cohort contained a lower proportion of STB (49.4%) and higher proportions of PS (34.2%) and BS (16.5%) than the derivation cohort (χ² = 5.90, P = 0.052), reflecting the intended case-mix difference for independent external testing.

Baseline demographic, clinical, laboratory, and quantitative lesion characteristics are summarized in **Table 1**. Age, sex, BMI, and symptom duration were comparable across the training, internal validation, and external validation sets. Marked differences emerged across the three diagnostic groups. Patients with STB were predominantly middle-aged with longer symptom duration and more frequent night sweats and weight loss, alongside lower serum albumin and prealbumin. Patients with PS showed the highest acute inflammatory markers, including CRP, ESR, NLR, and SII. Those with BS were younger, more frequently male, and more likely to reside in endemic regions. Quantitative lesion characteristics also differed: STB was associated with the largest lesion volume and the widest distribution of involved segments (median 2.0, IQR 1.0–3.0), whereas PS showed the highest T2W-FS entropy and STB the highest T1W GLCM contrast.

### Unimodal model performance

The performance of unimodal models is presented in **Table 2**. Among clinical-only models, LightGBM achieved the highest discriminative performance in both the internal and external validation sets, followed by XGBoost, random forest, and multinomial logistic regression. The quantitative lesion-only LightGBM model performed slightly worse than its clinical-only counterpart in external validation, suggesting that preoperative clinical and laboratory indicators alone carried marginally more generalizable discriminative information than radiomic features alone. Nevertheless, the lesion-only models captured unique anatomical patterns; for example, T1W GLCM contrast and T2W-FS GLRLM long-run emphasis were among the top discriminative features for STB, whereas T1W first-order skewness and GLCM dissimilarity were most informative for PS. The receiver operating characteristic curves comparing clinical-only and lesion-only models in the external validation set are shown in **Figure 2a**, and the calibration curves for the clinical-only LightGBM model are shown in **Figure 2f**.

**Table 2:** Discrimination and calibration performance of unimodal and multimodal models.

Model	Architecture	Internal validation AUC (95% CI)	External validation AUC (95% CI)	External validation accuracy (%)	Sensitivity (%)	Specificity (%)	PPV (%)	NPV (%)	Calibration brier
Clinical-only (C)									
Logistic Regression	Unimodal	0.724 (0.684–0.764)	0.712 (0.676–0.748)	74.2	68.5	78.4	72.6	80.2	0.238
Random Forest	Unimodal	0.763 (0.725–0.801)	0.748 (0.712–0.784)	76.5	72.8	80.2	75.4	82.6	0.226
XGBoost	Unimodal	0.782 (0.746–0.818)	0.765 (0.730–0.800)	78.2	75.2	82.1	77.8	84.5	0.218
LightGBM	Unimodal	0.791 (0.756–0.826)	0.772 (0.738–0.806)	79.5	76.8	83.4	79.2	85.8	0.214
Quantitative lesion-only (L)									
LightGBM	Unimodal	0.784 (0.748–0.820)	0.758 (0.723–0.793)	77.2	74.2	80.8	76.5	82.4	0.222
Multimodal fusion									
Logistic Regression	Early Fusion (EF)	0.798 (0.762–0.834)	0.805 (0.771–0.839)	78.5	77.2	81.8	78.6	84.2	0.182
Logistic Regression	Late Fusion (LF)	0.792 (0.756–0.828)	0.798 (0.764–0.832)	77.8	76.5	81.2	77.8	83.6	0.186
Random Forest	Early Fusion (EF)	0.822 (0.788–0.856)	0.815 (0.781–0.849)	80.5	78.8	84.2	80.5	86.2	0.172
Random Forest	Late Fusion (LF)	0.829 (0.795–0.863)	0.822 (0.788–0.856)	81.2	79.6	84.9	81.3	86.9	0.170
XGBoost	Early Fusion (EF)	0.838 (0.805–0.871)	0.834 (0.802–0.866)	81.8	80.2	85.6	82.1	87.5	0.168
XGBoost	Late Fusion (LF)	0.845 (0.812–0.878)	0.840 (0.809–0.871)	82.5	81.2	86.4	82.8	88.2	0.164
LightGBM	Early Fusion (EF)	0.852 (0.820–0.884)	0.845 (0.814–0.876)	82.8	81.5	86.8	83.2	88.5	0.162
LightGBM	Late Fusion (LF)	0.858 (0.826–0.890)	0.852 (0.822–0.882)	81.8	77.7	90.4	78.8	90.5	0.158

Macro-averaged metrics across the three diagnostic classes. AUC, area under the receiver operating characteristic curve; CI, confidence interval; PPV, positive predictive value; NPV, negative predictive value.

**Figure 2:**
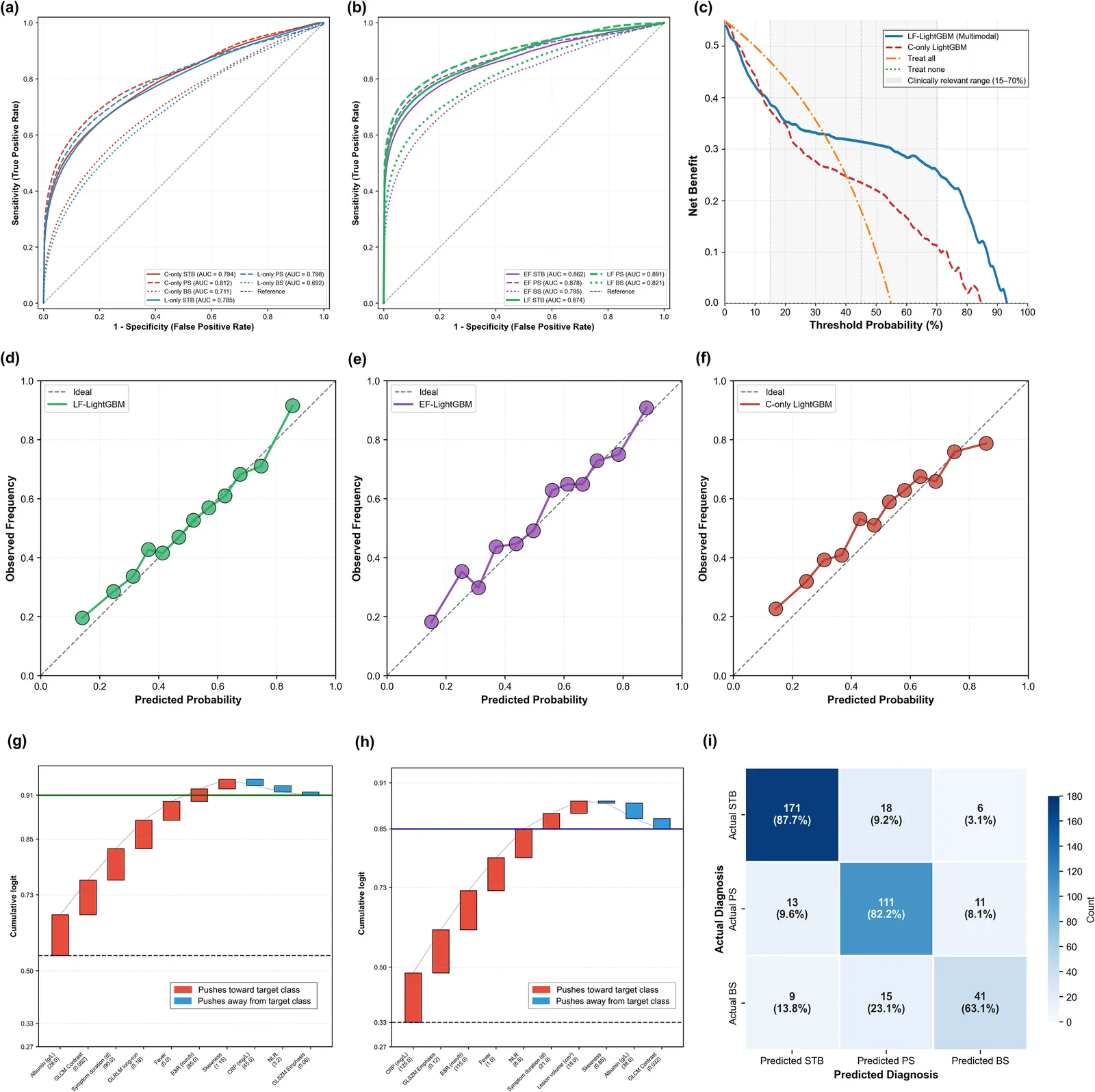
Model discrimination, calibration, clinical utility, and case-level explainability in the external validation set. **(a)** ROC curves of unimodal clinical-only (C-only) and quantitative lesion-only (L-only) models. **(b)** ROC curves of multimodal early fusion (EF) and late fusion (LF) models. **(c)** Decision curve analysis comparing the LF-LightGBM multimodal model, C-only LightGBM model, and treat-all/treat-none strategies. **(d–f)** Calibration curves for the LF-LightGBM, EF-LightGBM, and C-only LightGBM models. **(g, h)** SHAP waterfall decompositions for representative patients with spinal tuberculosis (STB; chronic course, hypoalbuminemia, caseous necrosis; g) and pyogenic spondylitis (PS; acute onset, high inflammation, abscess formation; h). **(i)** Confusion matrix for the LF-LightGBM model (n = 395).

### Multimodal fusion performance and incremental value

Multimodal fusion models consistently outperformed unimodal baselines across all algorithms (**Table 2**). In the external validation set, the late fusion LightGBM model achieved the highest overall discriminative performance (macro-averaged AUC 0.852, 95% CI 0.822–0.882), marginally surpassing the early fusion LightGBM model. For the two gradient-boosting algorithms (XGBoost and LightGBM), late fusion outperformed early fusion, probably because the meta-learner optimally weighted complementary probability distributions derived from independently trained unimodal models. For logistic regression, early fusion performed slightly better, consistent with its parametric nature. The ROC curves for all fusion models are illustrated in **Figure 2b**.

Integrating quantitative lesion features with clinical data yielded significant incremental discriminative value. Compared with the best clinical-only model, the late fusion LightGBM model showed a ΔAUC of 0.080 (95% CI 0.051–0.109; P < 0.001), an NRI of 0.398 (95% CI 0.268–0.528), and an IDI of 0.072 (95% CI 0.048–0.096). The relative utility at the 50% decision threshold was 0.18 versus 0.11 for the clinical-only model.

Class-specific performance is detailed in **Table 3** and illustrated in **Figure 2i**. The overall accuracy was 81.8%. Sensitivity was highest for STB and PS but lower for BS, reflecting class imbalance and the inherent overlap in imaging and clinical features between BS and early-stage STB. Specificity remained high across all classes. The one-vs-rest AUCs support the model’s utility for binary clinical decision-making scenarios such as ruling in or ruling out STB or PS.

**Table 3:** Class-specific classification performance of the LF-LightGBM model in the external validation set.

Metric	Spinal tuberculosis (STB)	Pyogenic spondylitis (PS)	Brucellar spondylitis (BS)	Macro-averaged
Confusion matrix				
True Positive, n	171	111	41	—
False Positive, n	22	33	17	—
False Negative, n	24	24	24	—
True Negative, n	178	227	313	—
Classification metrics				
Sensitivity, % (95% CI)	87.7 (82.8–92.1)	82.2 (75.4–88.2)	63.1 (51.2–73.8)	77.7
Specificity, % (95% CI)	89.0 (84.2–92.8)	87.3 (82.5–91.2)	94.8 (91.8–96.8)	90.4
Positive Predictive Value, % (95% CI)	88.6 (83.8–92.4)	77.1 (70.2–83.2)	70.7 (58.2–80.5)	78.8
Negative Predictive Value, % (95% CI)	88.1 (83.2–92.1)	90.4 (86.2–93.6)	92.9 (89.8–95.2)	90.5
F1-score	0.881	0.796	0.667	0.781
One-vs-rest AUC				
AUC (95% CI)	0.874 (0.842–0.906)	0.891 (0.862–0.920)	0.821 (0.756–0.886)	0.862

One-vs-rest AUCs compare each class against the other two pooled. The macro-averaged OvR AUC (0.862) is the arithmetic mean of the three class-specific OvR AUCs; it differs in definition from, and is not directly comparable to, the primary Hand and Till multiclass AUC (0.852) reported in **Table 2**. Numbers are per class in the one-vs-rest formulation. AUC, area under the receiver operating characteristic curve; CI, confidence interval; LF-LightGBM, late fusion LightGBM.

### Calibration and clinical utility

Calibration performance is summarized in **Table 2** and illustrated in **Figure 2**. The LF-LightGBM model demonstrated good calibration in the external validation set (Brier score 0.158), with a curve closely approximating the ideal diagonal (**Figure 2d**) and markedly outperforming the clinical-only model (**Figure 2f**). The EF-LightGBM model exhibited comparable calibration (**Figure 2e**), whereas the C-only models showed more pronounced miscalibration, particularly for the BS class.

Decision curve analysis demonstrated positive net benefit for the LF-LightGBM model across threshold probabilities from 15% to 70% (**Figure 2c**). Net benefit peaked at approximately 45%, where the model could prevent roughly 31 unnecessary biopsies per 100 patients while maintaining high sensitivity for true infection. Below 15%, a treat-all strategy dominated; above 70%, net benefit converged with a treat-none strategy.

### Model interpretability

SHAP analysis of the LF-LightGBM model in the external validation set revealed the complementary nature of clinical and radiomic predictors. The most influential features included six clinical variables (CRP, ESR, serum albumin, NLR, symptom duration, and endemic residence) and four quantitative lesion characteristics (T1W GLCM contrast, T2W-FS GLSZM large-area emphasis, T1W first-order skewness, and T2W-FS GLRLM long-run emphasis). The global feature importance ranking is presented in **Figure 3a**.

**Figure 3:**
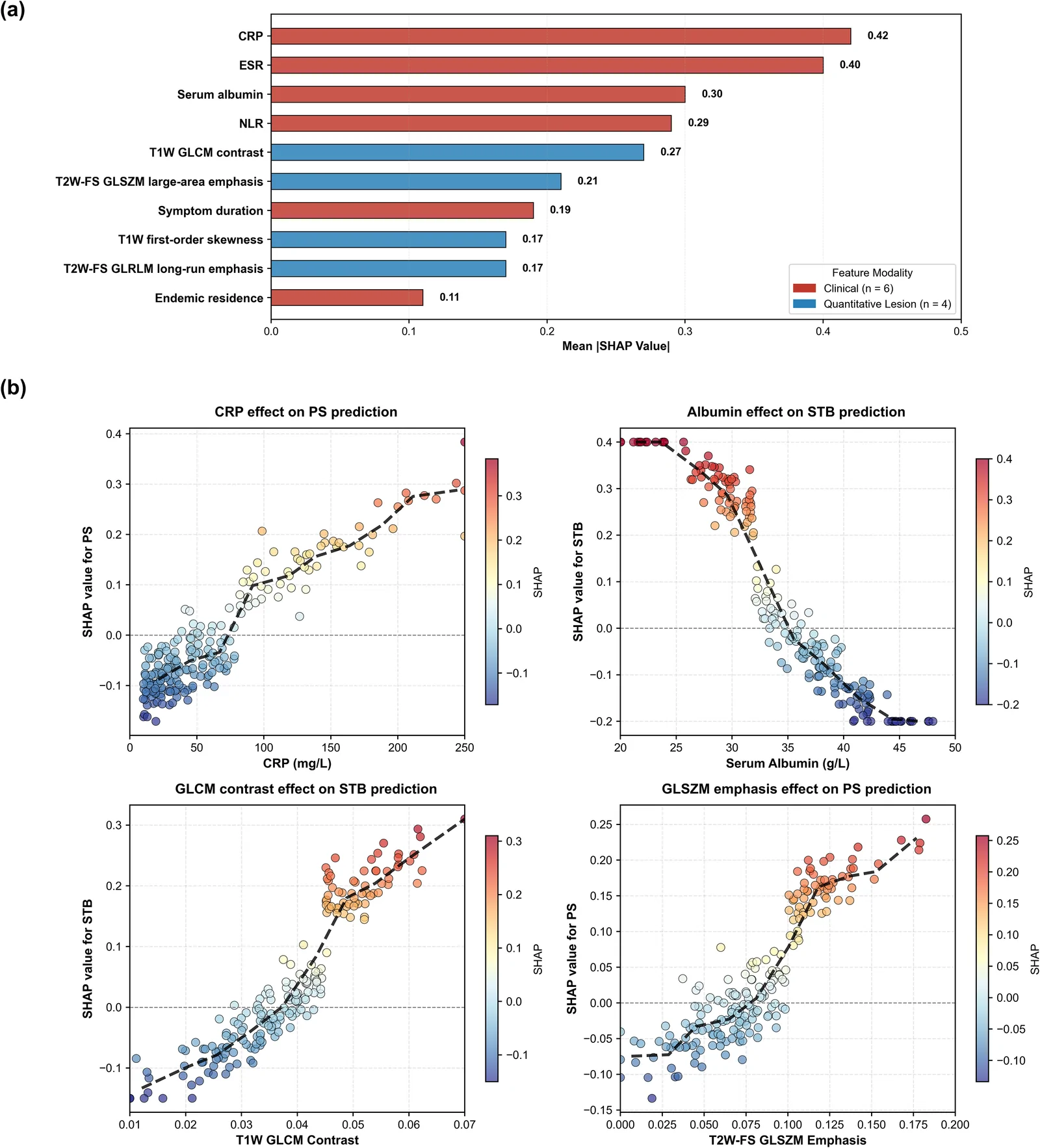
Global and feature-level interpretability of the LF-LightGBM model in the external validation set. **(a)** Global feature importance ranking by mean absolute SHAP value. **(b)** SHAP dependence plots showing the effects of top discriminative features on class-specific probabilities.

Class-specific contributions aligned with established pathophysiology. For STB, the strongest positive drivers were low serum albumin, prolonged symptom duration, and elevated T1W GLCM contrast, consistent with chronic nutritional depletion and the textural heterogeneity of caseous necrosis. For PS, high CRP, ESR, NLR, and T1W first-order skewness drove predictions, reflecting acute neutrophilic inflammation; CRP exhibited a sharp threshold effect above 80 mg/L. For BS, residence in an endemic region and low T2W-FS GLSZM large-area emphasis were key drivers, reflecting epidemiological exposure and subacute inflammation with relatively preserved vertebral architecture and small abscesses. SHAP dependence plots showed distinct directional effects and clinically actionable thresholds (**Figure 3b**), while waterfall decompositions for representative cases illustrated how each variable contributed to individual predictions (**Figure 2g, h**).

Correlating the top SHAP-ranked quantitative lesion features with their pathophysiological underpinnings connected abstract radiomic descriptors to recognizable pathological processes. T1W GLCM contrast reflected the interface between caseous necrosis and viable granulomatous tissue, T2W-FS GLSZM large-area emphasis corresponded to extensive fluid-signal abscesses, and T2W-FS GLRLM long-run emphasis was linked to contiguous inflammatory zones along the anterior longitudinal ligament.

### Sensitivity analyses

When SMOTE oversampling was applied to the training set, BS sensitivity improved from 63.1% to 70.8%; however, this gain was offset by a marginal decrease in macro-averaged AUC (−0.006) and a reduction in BS specificity (−2.7%), indicating a trade-off between minority-class recall and overall discriminative precision (**Table 4**). The class-weighted strategy was therefore retained as the primary analysis because it preserved the original data distribution and showed superior overall generalizability.

**Table 4:** Comparison of class-weighted loss and SMOTE oversampling strategies for the LF-LightGBM model in the external validation cohort (n = 395).

Metric	Class-weighted (primary)	SMOTE (sensitivity)	Difference
Macro-averaged AUC (95% CI)	0.852 (0.822–0.882)	0.846 (0.815–0.877)	−0.006
Overall accuracy (%)	81.8	81.0	−0.8
STB sensitivity (%)	87.7	86.2	−1.5
STB specificity (%)	89.0	87.8	−1.2
PS sensitivity (%)	82.2	80.5	−1.7
PS specificity (%)	87.3	86.2	−1.1
BS sensitivity (%)	63.1	70.8	+7.7
BS specificity (%)	94.8	92.1	−2.7
BS F1-score	0.667	0.712	+0.045
Macro F1-score	0.781	0.792	+0.011
Brier score	0.158	0.162	+0.004
Calibration slope	0.94	0.91	−0.03

In a secondary analysis, adding pathogen-specific serological tests to the primary model provided only modest incremental improvement (ΔAUC = 0.042), with notable gains limited to the one-vs-rest AUCs for STB and BS. This indicates that the primary model, built solely on routine preoperative clinical and imaging data, already achieves robust discrimination independently of serological confirmation.

Scanner heterogeneity analysis showed no significant associations between scanner manufacturer or field strength and the top 20 SHAP-ranked radiomic features after FDR correction, confirming cross-platform stability.

### *Post-hoc* power analysis for brucellar spondylitis

Given the relatively limited sample size of BS (n = 255, 13.9%), we conducted a *post-hoc* power analysis to assess the statistical adequacy of the cohort. Using the one-vs-rest AUC for BS in the external validation set (0.821, 95% CI 0.756–0.886) as the reference, the standard error was conservatively estimated at 0.033 using the Hanley-McNeil method. The power to detect an AUC exceeding 0.70 (a clinically acceptable minimum threshold) was 97.8%, whereas the power to detect an AUC exceeding 0.75 was 69.4%. These results indicate that the current sample size is sufficient to infer BS discriminatory performance at a good level (AUC > 0.70), though precision in estimating higher AUC values remains limited.

In terms of machine learning sample-size adequacy, the full multimodal feature set—as concatenated in the early fusion architecture—comprised 30 features (10 clinical and 20 lesion features). With 152 BS events in the training set, the events-per-variable (EPV) ratio was approximately 5.1, which falls below the traditional EPV ≥ 10 criterion. It should be noted that the late fusion architecture mitigates this concern by training independent unimodal sub-models and fusing only six probability inputs in the meta-learner, thereby reducing the effective dimensionality during training. Nevertheless, the BS subgroup remains at the boundary of sample-size sufficiency for feature selection, potentially limiting the model’s capacity to fully learn minority-class imaging patterns.

## Discussion

In this multicenter retrospective study, we developed and externally validated a preoperative multimodal machine learning model that integrates clinical-laboratory variables with non-contrast MRI radiomic features to differentiate spinal tuberculosis, pyogenic spondylitis, and brucellar spondylitis. The late fusion LightGBM model achieved a macro-averaged AUC of 0.852 in the independent external validation cohort, a significant improvement over both the clinical-only model (ΔAUC = 0.080) and the radiomic-only model. The addition of quantitative lesion features provided substantial incremental discriminative value, as shown by a net reclassification improvement of 0.398 and an integrated discrimination improvement of 0.072. The model showed good calibration, positive net benefit across a broad range of decision thresholds, and biologically interpretable predictions on SHAP analysis. These findings support the use of multimodal explainable AI as a clinically actionable decision-support tool for the differential diagnosis of infectious spondylitis.

The multimodal late fusion architecture outperformed unimodal baselines because clinical and imaging modalities provide complementary information. Preoperative clinical and laboratory variables, including CRP, ESR, serum albumin, and NLR, reflect the systemic inflammatory and nutritional state, whereas radiomic features quantify local tissue heterogeneity that mirrors distinct pathological processes. Late fusion independently trains unimodal models and then combines their probability distributions through a meta-learner (Teng et al., 2026); for the two gradient-boosting algorithms (XGBoost and LightGBM), this approach marginally outperformed early fusion (Yu et al., 2025), probably because preserving modality-specific feature representations allows the meta-learner to weight complementary confidence distributions optimally without the noise that arises from simple concatenation of high-dimensional features. For logistic regression, early fusion performed slightly better, consistent with the parametric nature of linear models. Beyond discrimination, SHAP analysis provided mechanistic interpretability consistent with established pathophysiology. For STB, the strongest predictors were low serum albumin, prolonged symptom duration, and elevated T1W GLCM contrast, reflecting chronic nutritional depletion and the textural heterogeneity of caseous necrosis. For PS, high CRP, elevated ESR, increased NLR, and T1W first-order skewness reflected acute neutrophilic inflammation; a CRP level above 80 mg/L acting as a decisive discriminator. For BS, residence in an endemic region and low T2W-FS GLSZM large-area emphasis were the key drivers, reflecting epidemiological exposure and subacute inflammation with relatively preserved vertebral architecture. These associations connect abstract radiomic descriptors to recognizable pathological processes, which should enhance clinician trust and facilitate clinical adoption.

Excluding pathogen-specific serological tests from the primary model was a deliberate methodological choice. T-SPOT.TB, IGRA, and Brucella SAT are themselves part of the reference standard for STB and BS (Freire et al., 2024; Feng et al., 2017; Wang H. et al., 2025); including them would have introduced data leakage and produced inflated, clinically unrealistic performance estimates. The secondary sensitivity analysis showed that adding these tests provided only a modest incremental gain (ΔAUC = 0.042), whereas the primary model, built with only routine preoperative clinical and imaging data, already achieved robust discrimination. The model can therefore provide immediate, independent diagnostic guidance when serological results are pending or unavailable. Previous studies on the differential diagnosis of infectious spondylitis have generally fallen into two categories: conventional biomarker-based approaches and unimodal machine learning models. Among conventional approaches, Liu et al (Liu et al., 2022). demonstrated that the neutrophil-to-lymphocyte ratio (NLR) could distinguish spinal tuberculosis from pyogenic spondylitis with an AUC of 0.87, yet this approach relied on a single hematological indicator and traditional statistical methods without integration of imaging information. More recently, Huang et al (Huang et al., 2024). developed a diagnostic model combining multiple machine learning algorithms to differentiate STB from PS, identifying platelet-to-neutrophil ratio as a key variable; however, their model was limited to binary classification and internal validation only, without external generalizability assessment. In radiomics-based studies, Wang et al. (Wang et al., 2023) extracted quantitative features from T2-weighted fat-suppression MRI to build random forest and support vector machine models for discriminating tuberculous from brucellar spondylitis, achieving AUCs of 0.950 and 0.904, respectively. While these results underscore the diagnostic potential of quantitative imaging, the study was confined to a single center, performed only binary classification, and did not incorporate clinical or laboratory variables. Collectively, these investigations have established important proof-of-concept for machine learning in infectious spondylitis, but they remain limited by unimodal designs, binary diagnostic frameworks, and the absence of rigorous external validation—gaps that our study addresses through a multimodal, multicenter, externally validated approach.

The limited sensitivity for BS (63.1%) warrants careful clinical consideration. This suboptimal performance stems primarily from two factors: (a) class imbalance, where BS represented only 13.9% of the cohort, inherently constraining the model’s learning capacity for minority-class patterns; and (b) clinical-radiological overlap, as subacute BS often presents with relatively preserved vertebral architecture and small abscesses, features that overlap with early-stage STB in the feature space.

Despite this limitation, the model maintains a high specificity for BS (94.8%) and a positive predictive value of 70.7% (**Table 3**). In brucellosis-endemic regions, a positive model prediction of BS may provide an important reference for clinicians considering targeted anti-brucellar therapy; conversely, although a negative prediction cannot entirely exclude BS, the negative predictive value of 92.9% may aid clinical decision-making when interpreted alongside epidemiological history. Importantly, the model is designed as a decision-support tool to augment, not replace, clinical judgment, microbiological culture, and serological testing. For patients with suspected BS, clinicians should integrate epidemiological exposure history (residence in endemic regions, contact with livestock, or consumption of unpasteurized dairy products) with the standard agglutination test (SAT) before finalizing treatment decisions. The SHAP analysis identified ‘residence in an endemic region’ as a key driver for BS classification, confirming that the model appropriately integrates clinically actionable epidemiological cues.

Future efforts to improve BS recognition should include: (a) prospective enrollment of additional BS cases to improve class balance; (b) implementation of cost-sensitive learning or threshold optimization strategies specifically tuned to minimize false negatives for BS; and (c) incorporation of contrast-enhanced MRI or diffusion-weighted imaging (DWI) sequences to capture BS-specific vascular and inflammatory patterns that are not evident on non-contrast sequences.

From a translational perspective, the model may serve as a decision-support tool in preoperative clinical workflows. Decision curve analysis showed that at a 45% probability threshold, the multimodal model could prevent approximately 31 unnecessary biopsies per 100 patients while maintaining high sensitivity for true infection. This triage function is especially valuable in resource-limited settings where advanced microbiological or molecular testing is unavailable or delayed. For patients with intermediate predicted probabilities, clinicians could prioritize empiric anti-tuberculosis therapy, broad-spectrum antibiotics, or targeted anti-brucellar regimens according to the dominant predicted class while awaiting definitive culture or molecular confirmation. Because the model relies on non-contrast MRI sequences, which are universally available and standardized, it should be broadly applicable without requiring gadolinium administration or specialized imaging protocols.

This study has several limitations. The retrospective design introduces selection bias because only surgical patients were included, and its performance in non-surgical patients with milder or medically managed disease is unknown. Class imbalance remains a key limitation: BS accounted for only 13.9% of the cohort (n = 255), resulting in a lower sensitivity of 63.1% compared with STB and PS. This reflects the real-world epidemiological distribution rather than selection bias, but it nonetheless restricts the model’s clinical utility for BS detection. *Post-hoc* power analysis confirmed adequate power (97.8%) to detect an AUC > 0.70 for BS, but the events-per-variable ratio (~5.1) in the training set fell below conventional recommendations. While SMOTE oversampling improved BS sensitivity to 70.8%, it concurrently reduced overall AUC and increased false-positive rates; consequently, we retained class-weighted loss as the primary strategy to preserve the original data distribution. Future prospective multicenter studies should prioritize enrolling additional BS patients to validate and enhance minority-class performance. Radiomic feature extraction required manual ROI segmentation, which is labor-intensive and operator-dependent; automated or semi-automated segmentation pipelines—such as those based on nnU-Net or transformer architectures—will be essential for scalable clinical deployment and will be integrated into the design of our forthcoming prospective studies. Because contrast-enhanced MRI was not routinely available across the retrospective multicenter cohort, we excluded these sequences, potentially omitting discriminative vascular and inflammatory enhancement patterns. All external validation cohorts were recruited in China, therefore, the model’s generalizability to other ethnicities, genetic backgrounds, and healthcare systems remains unknown. Fungal spondylitis and other rare infectious etiologies were not included in the diagnostic framework. Despite these limitations, this study addresses an important unmet clinical need for accurate preoperative differentiation of infectious spondylitis subtypes using routinely available data. By integrating non-contrast MRI radiomics with clinical-laboratory variables, the proposed approach offers a non-invasive, interpretable, and readily implementable decision-support framework that does not require contrast administration or invasive procedures. Future work should focus on prospective multicenter validation, automated segmentation, and expansion to non-surgical and diverse populations to fully realize its clinical utility.

## Conclusion

This multicenter study developed and validated a multimodal model for preoperative differentiation of spinal tuberculosis, pyogenic, and brucellar spondylitis. The late-fusion LightGBM model achieved robust external validation (AUC 0.852) with significant incremental value and clinically interpretable SHAP predictors. Despite good calibration, BS sensitivity remained limited. Prospective validation is warranted.

## Data Availability

The raw data supporting the conclusions of this article will be made available by the authors, without undue reservation.
